# Variability of Polyphenol Compounds in *Myrtus Communis* L. (Myrtaceae) Berries from Corsica

**DOI:** 10.3390/molecules15117849

**Published:** 2010-11-03

**Authors:** Toussaint Barboni, Magali Cannac, Lionel Massi, Yolanda Perez-Ramirez, Nathalie Chiaramonti

**Affiliations:** 1 SPE, U.M.R. C.N.R.S. 6134, University of Corsica, 20250 Corte, France; E-Mails: cannac@univ-corse.fr (M.C.); chiaramon@univ-corse.fr (N.C.); perez-ramirez@univ-corse.fr (Y.P.-R.); 2 Institut de Chimie de Nice (FR-CNRS 3037), Plateforme Technologique de Chimie-Spectrométrie de Masse, Faculté des sciences, Université de Nice Sophia-Antipolis, Parc Valrose, F-06108, Nice Cedex, France; E-Mail: Lionel.Massi@unice.fr (L.M.)

**Keywords:** flavonols, MS^n^, HPLC-DAD, biotic and abiotic factors

## Abstract

Polyphenol compounds were extracted from *Myrtus communis* L. berries (Myrtaceae) by maceration in 70% ethanol and analysed by HPLC-DAD and electrospray mass spectrometry. The *Myrtus *berries were collected at maturity from seven localities on the island of Corsica (France) and the sampling was carried out during three years. The polyphenol composition of Corsican *Myrtus* berries was characterized by two phenolic acids, four flavanols, three flavonols and five flavonol glycosides. The major compounds were myricetin-3-*O*-arabinoside and myricetin-3-*O*-galactoside. Principal components analysis (PCA) is applied to study the chemical composition and variability of myrtle berries alcoholic extracts from the seven localities. Canonical analysis and PCA data distinguishes two groups of myrtle berries characterized by different concentrations of polyphenols according to soil and years of harvest. The variations in the polyphenol concentration were due to biotic and abiotic factors.

## Introduction

*Myrtus communis* L. is a traditional plant from the Corsican coast. It is a shrub, one to three meters tall, with bright green leaves and white flowers during the blossoming season (June to July). The fruit is spherical in shape, dark blue to black in colour, edible but astringent, and grows in autumn [1]. Myrtle has some medicinal properties [2]; it is used as an antiseptic and disinfectant drug, but also for its balsamic properties. The aromatic and medicinal qualities of *M. communis* contribute to its use in pharmaceutical, cosmetics and food products [3,6]. Myrtle berries are used to produce myrtle liqueur, eau-de-vie and a wine characteristic of Mediterranean areas [4]. The last 15 years have seen a growth in worldwide concern food quality, safety and geographical origin of the species [5]. 

Flavonoids are divided into several classes, which include flavonols, flavones, flavanones, flavanols, isoflavones, dihydroflavonols and chalcones and anthocyanidins [6]. The polyphenol components of leaves and berries from *M. communis* have been studied [5,7,8,9,10,11,12,13,14]. The first studies on the polyphenolic compounds from *M. communis* were undertaken by El-Sissi and El-Ansary [15]. Their study related to the analysis of flavonoids in myrtle leaves. In 1987, Diaz and Abeger [16] analysed the composition of flavonoids and phenolic acids in leaves of *Myrtus *spp*, *reporting the presence of the compounds gallic acid, ellagic acid, quercetin and patuletin. More recently, Martin *et al.* [11] studied the polyphenols in *M. communis* and they showed an important contribution of myricetin, hesperidin and esculin to the composition. Romani *et al.* [12] studied the composition of polyphenols extracted from leaves with 70% ethanol. The main compounds in this case were three phenolics, three flavanols and four flavonol glycosides. Recently, Montoro *et al*. [17,18] have studied the stability and antioxidant activity of polyphenols in alcoholic extracts of *M. communis* berries used for the preparation of liqueur in Sardinia and Italy. They showed the presence of six flavonoids and eight anthocyanins by HPLC-UV-VIS and identification was done by LC-ESI-MS. Degradation of anthocyan content and antioxidant activity was observed in myrtle liquor after a storage period of three months. The influence of maceration period on the polyphenolic composition of hydroalcoholic extracts obtained from different selections of Italian berries was also reported [19,20]*. *Piras *et al. *[5] showed a variation in anthocyanins, flavonols and α-tocopherol from alcoholic extracts of myrtle berries obtained from seven different sites. PCA determined the dissimilarities between samples. 

The aims of this work were: i) to characterize the polyphenol composition of Corsican berry extracts, and ii) to study the variability of different localities and variations during a period of three years within the framework of a valorisation of myrtle products in relation to the quality of myrtle liquor from Corsica. 

## Results and Discussion

### Polyphenolic composition of M. communis berries

**Figure 1:**
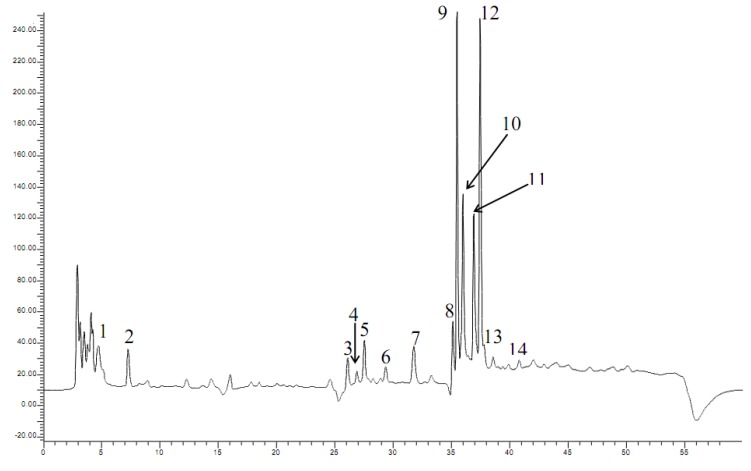
Example of the chromatographic profile of polyphenols from *Myrtus communis *obtained at 280 nm.

Fourteen phenolic compounds were identified in the *Myrtus communis *L. berry extracts by LC-MS/MS and HPLC-DAD. Two phenolic acids **1–2**, four flavanols **3–6**, five flavonol glycosides **7–11**, and three flavonols **12–14** were present (Figure 1). Myricetin and glycosides derivatives were the major constituents from myrtle berries (Table 1).

Flavonol glycosides were the major compounds found in the myrtle extracts. They represented 58% of the total amount of phenolic compounds identified at 280 nm by HPLC-UV. Myricetin-3-*O*-arabinoside was the major constituent (106.6 to 1435.9 mg/100g_dw_). These flavonols compounds are present in most fruits and plants, and are also the most studied [21]*.* Their anti-cancer action is also acknowledged [22,23,24].

The major component was myricetin (207.8 to 1053.6 mg/100g_dw_). (-) Epigallocatechin, with a concentration range between 124.0 to 952.9 mg/100g_dw_, was the main constituent from the flavanol family. Phenolic acids were present at low concentrations. The major compounds were previously identified in the literature [12,17,18,25]. Romani *et al.* [12] identified caffeic acid, in addition to the compounds identified in our samples. Montoro *et al.* [17,18] showed the presence of six flavonoids. In the berry extracts from *Myrtus *we identified quercetin and kaempferol that aren’t present in other berry extracts.

**Table 1:** Polyphenol concentrations from all sites during three years.

**Number**	**Compounds**	**Site 1**			**Site 2**			**Site 3**			**Site4**			**Site 5**			**Site 6**			**Site 7**		
	(mg/100g_dw_ of berries)	2003	2004	2005	2003	2004	2005	2003	2004	2005	2003	2004	2005	2003	2004	2005	2003	2004	2005	2003	2004	2005
																						
**1**	Gallic acid	61.5 ± 2.1	38.0 ± 1.5	80.9 ± 3.8	167.6 ± 6.2	264.4 ± 8.5	82.7 ± 4.1	42.5 ± 1.4	112.0 ± 5.8	76.8 ± 3.4	80.1 ± 4.1	58.0 ± 3.5	28.5 ± 0.8	14.2 ± 0.4	18.6 ± 0.5	14.6 ± 0.6	23.1 ± 0.8	113.3 ± 5.9	45.2 ± 2.5	15.3 ± 0.8	30.2 ± 1.1	35.1 ± 2.0
**2**	Ellagic acid	56.9 ± 2.8	34.3 ± 1.4	75.1 ± 3.1	138.5 ± 5.2	195.3 ± 9.5	119.9 ± 5.9	62.5 ± 3.2	78.9 ± 4.2	103.6 ± 5.3	98.5 ± 4.5	86.3 ± 3.2	25.6 ± 0.9	45.5 ± 1.1	44.9 ± 1.8	40.2 ± 1.8	44.3 ± 2.2	90.4 ± 4.4	40.9 ± 2.9	35.3 ± 1.4	38.1 ± 2.2	20.8 ± 1.1
**3**	(+) Catechin	191.3 ± 8.2	130.9 ± 5.2	240.5 ± 9.9	149.6 ± 6.6	115.6 ± 4.1	197.6 ± 9.8	70.1 ± 5.5	58.2 ± 2.2	208.4 ± 9.9	87.2 ± 4.4	109.2 ± 5.8	83.4 ± 4.1	108.1 ± 5.1	147.4 ± 6.8	113.7 ± 5.9	186.8 ± 8.8	95.9 ± 4.1	123.7 ± 4.9	90.9 ± 3.2	107.3 ± 4.4	79.7 ± 3.3
**4**	(-) Epicatechin-3- *O*-gallate	75.3 ± 1.9	45.5 ± 2.8	98.7 ± 3.6	29.4 ± 1.4	1.3 ± 0.2	55.8 ± 1.1	37.5 ± 2.2	93.8 ± 4.4	74.4 ± 3.2	17.2 ± 3.3	63.7 ± 4.5	43.9 ± 3.8	18.8 ± 1.5	20.5 ± 2.2	17.5 ± 4.5	22.1 ± 2.1	26.5 ± 1.2	15.5 ± 0.8	65.8 ± 4.4	40.7 ± 3.8	33.0 ± 1.4
**5**	(-) Epigallocatechin	742.1 ± 32.4	483.1 ± 20.8	952.9 ± 54.4	507.0 ± 25.5	290.4 ± 15.2	805.5 ± 40.8	263.6 ± 16.5	124.0 ± 6.2	835.8 ± 36.2	295.8 ± 15.8	418.7 ± 24.8	237.5 ± 12.8	241.4 ± 9.6	297.4 ± 15.9	239.8 ± 12.8	353.3 ± 18.7	209.9 ± 9.2	222.3 ± 11.9	321.7 ± 15.1	272.0 ± 12.1	195.4 ± 20.9
**6**	(-) Epigallocatechin-3- *O*-gallate	457.7 ± 25.2	340.2 ± 13.2	555.0 ± 25.8	370.7 ± 16.2	263.0 ± 15.5	536.3 ± 14.5	136.7 ± 5.9	96.5 ± 4.8	526.3 ± 25.5	181.3 ± 8.9	311.4 ± 13.9	104.3 ± 5.4	71.6 ± 3.2	99.5 ± 4.2	76.1 ± 5.7	127.3 ± 6.7	165.2 ± 6.4	135.7 ± 7.8	151.0 ± 7.9	143.3 ± 6.9	80.9 ± 2.5
**7**	Myricetin-3- *O*-galactoside	969.5 ± 52.1	771.0 ± 36.2	1138.3 ± 58.7	795.4 ± 42.1	604.8 ± 36.7	1021.9 ± 54.3	318.3 ± 16.2	330.1 ± 19.6	1012.4 ± 52.2	372.7 ± 20.2	644.2 ± 32.8	198.3 ± 9.5	133.6 ± 6.4	172.5 ± 9.5	136.2 ± 6.7	211.5 ± 9.9	316.7 ± 13.8	142.9 ± 6.8	244.6 ± 10.5	193.8 ± 14.5	175.1 ± 11.5
**8**	Myricetin-3- *O*-rhamnoside	451.3 ± 21.2	531.2 ± 36.2	390.4 ± 22.1	354.2 ± 15.6	286.7 ± 12.4	328.1 ± 16.7	205.2 ± 12.1	158.6 ± 9.8	362.7 ± 16.4	222.2 ± 11.8	270.4 ± 11.2	226.5 ± 12.2	155.0 ± 13.3	165.2 ± 20.9	142.5 ± 14.4	175.4 ± 16.6	175.5 ± 7.2	130.9 ± 8.9	279.4 ± 14.4	205.2 ± 20.8	200.0 ± 9.4
**9**	Myricetin-3- *O*-arabinoside	1290.3 ± 53.2	1435.9 ± 76.5	1181.6 ± 56.2	1120.4 ± 55.1	980.2 ± 48.9	1063.7 ± 48.7	393.1 ± 22.1	363.4 ± 12.2	1150.6 ± 48.9	530.8 ± 19.7	758.9 ± 31.2	538.3 ± 24.8	139.7 ± 8.2	176.0 ± 8.1	140.5 ± 7.2	212.4 ± 9.8	411.9 ± 20.1	106.6 ± 5.7	788.3 ± 45.2	447.4 ± 17.8	413.3 ± 19.4
**10**	Quercetin-3- *O*-glucoside	515.9 ± 26.2	316.8 ± 16.5	678.6 ± 35.4	769.6 ± 42.0	905.1 ± 46.5	840.8 ± 44.1	245.9 ± 16.2	322.6 ± 14.2	760.7 ± 36.2	420.8 ± 20.5	522.2 ± 23.9	159.1 ± 6.1	83.5 ± 4.8	96.1 ± 6.9	79.9 ± 5.2	108.8 ± 5.6	398.0 ± 16.4	118.8 ± 6.2	217.2 ± 9.9	168.0 ± 8.1	1130.0 ± 25.9
**11**	Quercetin-3- *O*-rhamnoside	646.8 ± 35.2	471.6 ± 25.4	792.3 ± 41.2	950.8 ± 51.0	1140.6 ± 53.3	931.7 ± 46.5	311.2 ± 15.6	417.5 ± 21.4	854.7 ± 45.9	525.6 ± 21.5	596.3 ± 28.9	214.7 ± 13.6	247.3 ± 12.8	327.6 ± 18.8	255.8 ± 11.4	407.9 ± 22.9	522.1 ± 26.2	269.7 ± 18.2	284.4 ± 21.8	277.0 ± 20.9	179.8 ± 9.4
**12**	Myricetin	762.0 ± 36.6	406.4 ± 22.2	1048.5 ± 56.5	746.4 ± 41.0	671.3 ± 36.6	1053.6 ± 51.1	277.9 ± 15.4	246.2 ± 14.2	1030.8 ± 52.2	439.5 ± 6.9	572.0 ± 22.2	265.1 ± 13.5	462.4 ± 22.1	658.3 ± 32.2	498.7 ± 26.2	854.2 ± 42.2	423.9 ± 32.1	462.0 ± 21.2	379.9 ± 16.2	420.9 ± 21.2	207.8 ± 5.6
**13**	Quercetin	448.7 ± 22.1	223.8 ± 12.2	629.7 ± 23.4	397.0 ± 21.8	376.6 ± 25.9	479.0 ± 25.5	156.5 ± 9.8	135.5 ± 8.3	517.8 ± 26.9	207.8 ± 11.2	205.4 ± 12.2	173.3 ± 9.8	183.5 ± 10.5	246.3 ± 15.2	191.3 ± 10.2	309.2 ± 15.6	182.6 ± 8.1	168.9 ± 10.2	253.5 ± 16.2	211.2 ± 14.2	133.2 ± 12.2
**14**	Kaempferol	182.1 ± 11.4	180.9 ± 14.5	265.4 ± 24.1	262.3 ± 22.9	334.8 ± 26.4	240.8 ± 26.4	77.7 ± 4.5	113.4 ± 5.4	241.5 ± 12.2	131.2 ± 4.5	115.3 ± 4.5	59.6 ± 2.2	41.8 ± 1.8	53.9 ± 1.8	42.6 ± 1.5	66.0 ± 2.1	137.7 ± 8.9	65.5 ± 1.2	87.4 ± 4.9	76.4 ± 3.8	45.7 ± 1.4
t	Total	6450.7 ± 336.4	5103.7 ± 284.6	7648.0 ± 418.2	6526.5 ± 352.6	6362.5 ± 339.6	7341.1 ± 375.4	2524.6 ± 146.6	2633.1 ± 132.7	7333.8 ± 374.6	3527.9 ± 157.3	4506.9 ± 222.6	2279.4 ± 119.5	2114.5 ± 101.0	1948.9 ± 134.8	2498.3 ± 144.7	2548.2 ± 156.0	3194.6 ± 171.3	917.4 ± 104.7	1359.8 ± 171.9	3301.9 ± 151.8	5995.3 ± 126.0

### Effects of localities of origin of M. Communis berries on polyphenol compound compositions

**Figure 2:**
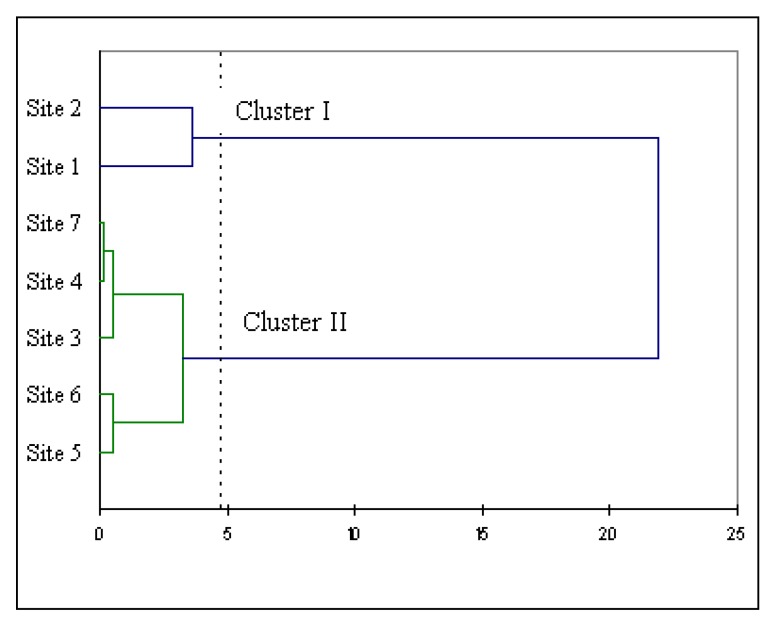
Dendrogram obtained from the cluster analysis of composition of polyphenols from seven localities on Corsica Island. Samples are clustered using Ward's technique with an Euclidean distance measure.

The localities of origin have a significant effect on the polyphenol composition of myrtle extracts (Table 2). The total concentration was not significantly different between localities 1 and 2 (ANOVA, p > 0.05). Their concentrations, measured at between 6,400.8 ± 281.4 to 6,743.3 ± 241.8 mg/100g_dw_, were the highest observed among all the sites. Similarly, localities 5 and 6 have total concentrations of polyphenols which were not significantly different (ANOVA, p > 0.05). The other localities were significantly different (ANOVA, p < 0.05). To synthesize the polyphenolic compositions data, Canonical Analysis (CA) was applied to examine the relative distribution of localities according to polyphenol compounds (Figure 2). 

**Table 2:** Means of polyphenol concentrations in *Myrtus communis* L. berries extracts (mg/100g_dw_ of berries) during three years (a,b,c,d,e,f: significant difference between localities and polyphenols concentrations, Tukey test, p < 0.05).

Components	Site 1	Site 2	Site 3	Site 4	Site 5	Site 6	Site 7
Phenolic acids	115.6 * ± *4.9^a^	322.8 * ± *13.1^b^	158.8 * ± *17.8^c^	125.7 * ± *5.7^ae^	67.9 * ± *2.1^d^	126.6 * ± *6.2^ae^	140.3 * ± *12.9^e^
Flavanols	1042.2 * ± *67.8^a^	868.6 * ± *50.3^b^	670.3 * ± *45.8^c^	522.3 * ± *107.5^c,d,f^	426.6 * ± *25.9^d^	320.0 * ± *28.7^e^	543.5 * ± *28.6^f^
Flavonol glycosides	3860.5 * ± *197.4^a^	4031.3 * ± *204.6^a^	2402.3 * ± *119.7^b^	1867.0 * ± *96.0^c^	995.1 * ± *58.0^d^	1254.0 * ± *50.8^e^	2282.8 * ± *86.5^c^
Flavonols	1382.5 * ± *74.3^a^	1520.6 * ± *87.8^a^	932.4 * ± *49.6^b^	723.1 * ± *29.0^c^	697.7 * ± *44.3^c^	519.6 * ± *43.5^d^	785.8 * ± *31.9^b^
Total polyphenols	6400.8 * ± *281.4^a^	6743.3 * ± *241.8^a^	4163.3 * ± *178.0^b^	3237.3 * ± *156.5^c^	2186.7 * ± *111.3^d^	2219.7 * ± *134.0^d^	3751.7 * ± *129.2^e^

The cluster analysis suggested the existence of groups based on the polyphenol concentrations. The first group was constituted by localities 1 and 2, and the second group by the other localities. The general structure of the dendrogram confirms the ANOVA results and suggests the existence of two main clusters. The same result was reported by Piras *et al* [5] who showed that the PCA of ToF-SIMS data from alcoholic extracts of myrtle berries distinguishes two groups characterized by a different concentration of anthocyanins, flavonols and α-tocopherol. 

**Figure 3:**
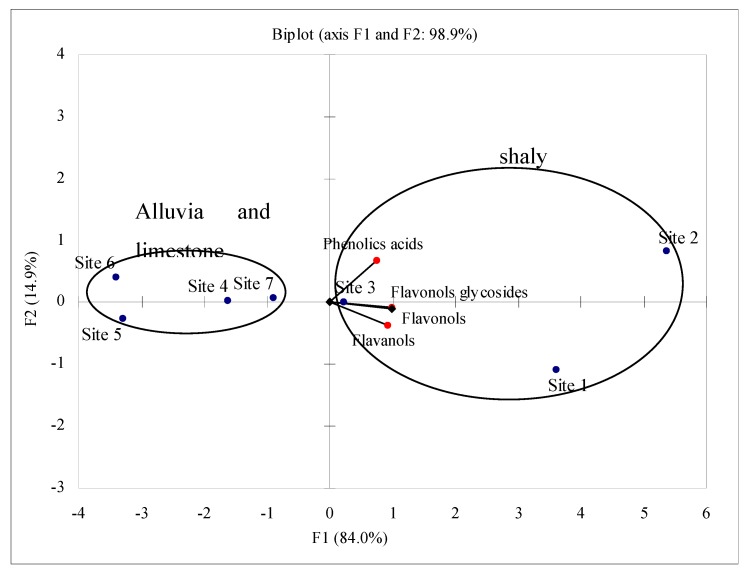
PCA biplot of correlations between sites and polyphenol compounds.

The two PCA axes explained 84.0% and 14.9% of the variance respectively. The first PCA axis was positively correlated with all polyphenol compounds. The second PCA axis was positively correlated with phenolic acids and negatively with flavanols, flavonol glycosides and flavonols. PCA offered a representation of the distribution of polyphenol compounds families from different localities. As shown in Figure 3, the first axis explained 84.0% of the variance and showed that the polyphenols concentration was more important from localities 1, 2 and 3, corresponding to shaly soil. In the literature data, a variation of anthocyanin derivatives in myrtle berries collected in different geographical areas in Italy [18] and Sardinia [5] were observed. Moreover, localities 4–7 showed a lower concentration of polyphenols in alluvia and limestone soil as compared to shaly soil. The total amount of polyphenols was thus more important in the shaly soil sites. 

### Effect of the meteorological conditions on the polyphenol compositions of M. communis berries

**Figure 4:**
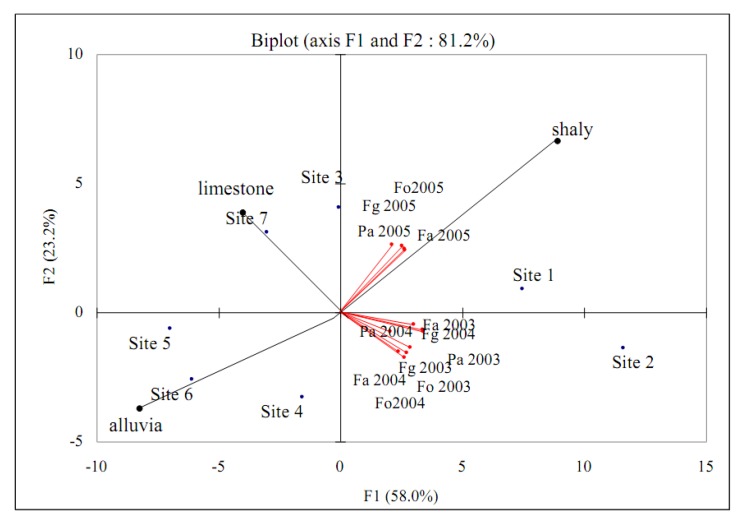
PCA biplot of correlations between sites, years of harvest and polyphenols compounds (Fg: Flavonols glycosides, Fa: Flavanols, Fo: Flavonols and Pa: Phenolics acids).

An important variability between polyphenols concentration and year of harvest was observed (Table 3). The polyphenol concentration was significantly different (ANOVA, p < 0.05) from one year to another during the three years of harvest examined. This variability is not related to the soil and origin of the *M. communis* berries. We noticed however an exception to this general behaviour for flavanols and flavonol glycosides, whose concentration was not significantly different (ANOVA, p > 0.05) for localities 5 and 2, respectively. The two PCA axes explained 58.0% and 23.2% of the variance, respectively (Figure 4). The first PCA axis was positively correlated with all polyphenol compounds and shaly soil and negatively correlated with limestone and alluvia. 

**Table 3:** Polyphenol concentrations in *Myrtus communis* L. berry extracts (mg/100g_dw_ of berries) during three years (a,b,c: significant difference between year and polyphenols concentrations, Tukey test, p < 0.05).

		Site 1	Site 2	Site 3	Site 4	Site 5	Site 6	Site 7
Phenolic acids	2003	118.4 * ± *4.9^a^	306.1 * ± *11.4^a^	105.0 * ± *4.6^b^	178.6 * ± *8.6^a^	59.4 * ± *1.5^a^	118.7 * ± *3.0^a^	75.0 * ± *2.2^a^
	2004	72.4 * ± *2.9^b^	459.7 * ± *18.0^b^	191.0 * ± *10.0^a^	144.3 * ± *6.7^b^	85.9 * ± *2.3^b^	203.6 * ± *10.3^b^	136.9 * ± *3.3^b^
	2005	156.1 * ± *6.9^c^	202.6 * ± *10.0^c^	180.4 * ± *8.9^a^	54.1 * ± *1.7^c^	58.3 * ± *2.4^a^	57.4 * ± *5.4^c^	209.0 * ± *3.1^c^
Flavanols	2003	1065.6 * ± *67.7^a^	824.4 * ± *49.7^a^	433.8 * ± *30.1^a^	498.6 * ± *32.4^a^	445.0 * ± *19.6^a^	398.5 * ± *28.9^a^	264.7 * ± *30.6^a^
	2004	693.8 * ± *42.0^b^	602.7 * ± *35.0^b^	354.9 * ± *17.6^b^	677.9 * ± *49.0^b^	401.4 * ± *29.1^a^	422.6 * ± *36.3^a^	465.8 * ± *27.2^b^
	2005	1367.2 * ± *93.7^c^	1178.8 * ± *66.2^c^	1222.3 * ± *74.8^c^	390.4 * ± *26.1^c^	433.5 * ± *28.9^a^	139.0 * ± *20.9^b^	900.0 * ± *28.1^c^
Flavonol glycosides	2003	3873.8 * ± *187.9^a,b^	3990.4 * ± *205.8^a^	1473.7 * ± *82.2^a^	2072.2 * ± *93.7^a^	817.1 * ± *45.5^a^	1376.9 * ± *41.9^a^	797.2 * ± *101.8^a^
	2004	3526.5 * ± *190.8^a^	3917.3 * ± *197.8^a^	1592.2 * ± *77.2^a^	2492.0 * ± *128.0^b^	767.0 * ± *64.2^b^	1824.2 * ± *64.8^b^	1938.4 * ± *82.1^b^
	2005	4181.1 * ± *213.6^b^	4186.2 * ± *210.3^a^	4141.1 * ± *199.6^b^	1336.8 * ± *66.2^c^	1401.2 * ± *64.2^c^	560.4 * ± *45.8^c^	3612.7 * ± *75.6^c^
Flavonols	2003	1392.8 * ± *70.1^a^	1405.6 * ± *85.7^a^	512.1 * ± *29.7^a^	778.5 * ± *22.6^a^	793.0 * ± *34.4^b^	654.1 * ± *37.9^a^	223.0 * ± *37.3^a^
	2004	811.1 * ± *48.9^b^	1382.8 * ± *88.9^a^	495.1 * ± *27.9^a^	892.7 * ± *38.9^b^	694.6 * ± *49.2^a^	744.2 * ± *59.9^b^	760.8 * ± *39.2^b^
	2005	1943.6 * ± *104.0^c^	1773.4 * ± *88.9^b^	1790.0 * ± *91.3^b^	498.1 * ± *25.5^c^	605.4 * ± *49.2^a^	160.6 * ± *32.6^c^	1373.7 * ± *19.2^c^

In the literature, Piras *et al.* [5] showed that the differences were not necessarily ascribable to geoclimatic factors, and genetic and/or environmental factors could be considered to affect the quantitative composition of myrtle berry extracts and irrigation affected the concentration of many compounds. As shown in Figure 4, the year 2005 presented the highest polyphenol concentration, while years 2003 and 2004 presented lower concentrations. We noticed however that along the three years of harvest, the concentrations were more important when berries were grown on shaly soils. Moreover, the biotic and abiotic factors induced the same differences in polyphenol concentrations (Figure 4). These variations can impact the quality of myrtle-derived products (myrtle liquor). 

## Conclusions

The major constituents of *Myrtus communis*. L. leaf extracts were myricetin-3-*O*-arabinoside and myricetin-3-*O*-galactoside. We showed that there are variations in the concentrations of polyphenols according to the sampling sites and years of harvest. The polyphenol concentrations were more important on sites characterized by shaly soils. These concentrations were low when the soil was constituted by alluvia. These levels vary according to biotic and abiotic factors and consequently year to year, in line with the notion of "vintage" as it refers to myrtle liquor and wine products. 

## Experimental

### Sampling and reagents

Samples were collected in seven stations spread over the whole island, under various environmental conditions (pedoclimatic conditions; Table 4). We collected 400 grams of berries *M*. *communis* for each locality on the same individual tree during three years in the same vegetative stage (November, 15^th^). November corresponds to the maturity of the berries and thus their harvest for the preparation of wines and liqueurs. One hundred grams were used for the extraction of polyphenol compounds. All extractions were made in triplicate (3 × 100 g). Sampling should allow the detection of a specific intra-variability that can lead to a possible chemical polymorphism. The berries were lyophilised and extracted with a solution of ethanol (500 mL at 70%) during one day at room temperature. After filtration on a Buchner funnel and extraction with ethyl acetate (3 × 50 mL; Aldrich > 99.9%), the solvent was evaporated with a rotary evaporator at 30 °C under vacuum. The extracts were combined and evaporated. The residue obtained is dissolved in 10 mL of methanol (Aldrich > 99.9%), and then filtered through a 0.45 µm membrane (Phenomenex, France). 

**Table 4:** Main characteristics of the studied sites and main parameters concerning *Myrtus* berry extracts.

	Site 1	Site 2	Site 3	Site 4	Site 5	Site 6	Site 7
Localities	Canari	Bastia	Agriate	Corte	Ajaccio	Morta	Bonifacio
Coordinates	09°19'56''	09°26'49''	09°18'15''	09°09'05''	08°44'06''	09°19'31''	09°09'20''
	42°50'43''	42°41'40''	42°40'50''	42°18'15''	41°55'14''	42°00'38''	41°23'15''
Soil type	shaly	shaly	shaly	alluvia	alluvia	alluvia	limestone

### HPLC-DAD conditions

Chromatographic equipment consisted of a Perkin Elmer LC 200 Series Model liquid chromatograph, an injection valve with a 20 µL sample loop, a degasser system, a binary pump and a diode-array detector (DAD). The whole chromatographic system was controlled by Totalchrom software running on computer. Chromatograms and spectra were obtained. An Aqua RP-C18 column (Phenomenex) (250 mm × 4,6 mm, 5 µm particle size) and an Aqua C_18_ precolumn (Phenomenex) were used at ambient temperature. The solvent system was: solvent A, 0.1% acetic acid and solvent B, acetonitrile. The column was equilibrated in 90% A-10% B, and elution was carried out with the following gradient: 90–80% A (0–15 min); 80–60% A (15–25 min); 60–40% A (25–35 min); 40–30% A (35–45 min); 0% A (45–55 min). Detection was carried out at 280 nm.

### Quantification of phenolic compounds

The polyphenols were quantified using external standards which were purchased from Extrasynthese (Geney, France). Five injections were made for each level, and a weighed linear regression was generated. The calibration curve with the external standard was obtained using concentrations with respect to the area obtained from the integration of peaks. The relation between variables was analysed using linear simple correlation.

### MS^n^ standard analysis

MS and MS*n *analysis were carried out on a Finnigan LCQ Classic ion trap mass spectrometer (Thermo, formerly Finnigan-MAT, San Jose, CA, USA) equipped with an APCI interface and an ion trap mass analyzer. The software used is Xcalibur. A syringe pump was used for the direct infusions of reference compound solutions, at a flow rate set at 5 µL/min in an HPLC flow (500 µL/min) composed of acetonitrile and MilliQ (18.2 MΩ) water with 0.1% of acetic acid through an HPLC tee. Solutions of standards were prepared at a concentration of 0.1 mg/mL. In other cases, the reference compounds were directly diluted in acetonitrile (LC/MS grade ROTISOLV 99.95% purchased from Carl Roth, Karlsruhe)

The operating parameters were as follows: damping gas, helium (He); nebulizing gas, nitrogen (N_2_); maximum injection time was set at 50 ms; the number of microscans was set at 3. For APCI in negative and positive mode, the source parameters were set as follow: discharge current, 5 µA; capillary temperature 200 °C; sheath gas (N_2_), 90 (arbitrary units); auxiliary gas, 25 (arbitrary units). In order to maximize the response for each reference compound, the voltages on the lenses (capillary voltage, tube lens offset, second and first octapole offset, interoctapole lense) were optimized using the automated LCQ TunePlus function of Xcalibur software. For MS/MS experiments, CID was carried out using Helium as collision gas. The collision energy are reported as a percentage of the maximum 5 V_p-p_ normalized for the parent ion m/z (NCE: Normalized Collision Energy) [26]. Collision energy for CID was optimized between 25% and 46% of maximum, and the isolation with of precursor ions was 1.5 amu.

### Data analysis

The significant difference for statistical analysis was determined by one-way analysis of variance (ANOVA). ANOVA was used when the application conditions were satisfied, *i.e.* normal distribution of treatment group means and homogeneity of variances between means, using the Shapiro-Wilk test and Bartlett test, respectively. Differences were considered to be significant when p < 0.05. When a difference was found, Tukey's HSD post-hoc comparison technique was used to determine which plots were different. The correlations between the polyphenols concentration from localities and extracted berries of *M. communis* were established using PCA on a matrix formed by the association of different classes of polyphenols compounds. CA produced a dendrogram (tree) using Ward’s method of hierarchical clustering, based on the Euclidean distance between pairs of sample localities. The Statistical Graphics Corporation's® "Statgraphics for Windows" software package was used for these various tests, together with Stat R version 2.6.1. PCA and CA are data mining tools that are useful for providing unsupervised visual classification of multivariate data like GC data [27].
